# Physico-Chemical Properties of Inorganic NPs Influence the Absorption Rate of Aquatic Mosses Reducing Cytotoxicity on Intestinal Epithelial Barrier Model

**DOI:** 10.3390/molecules26102885

**Published:** 2021-05-13

**Authors:** Valeria De Matteis, Makarena Rojas, Mariafrancesca Cascione, Stefano Mazzotta, Gian Pietro Di Sansebastiano, Rosaria Rinaldi

**Affiliations:** 1 Department of Mathematics and Physics “Ennio De Giorgi”, University of Salento, Via Arnesano, 73100 Lecce (LE), Italy; mariafrancesca.cascione@unisalento.it (M.C.); ross.rinaldi@unisalento.it (R.R.); 2 Department of Biological and Environmental Sciences and Technologies (DiSTeBA), University of Salento, 73100 Lecce (LE), Italy; makarena.rojas@unisalento.it (M.R.); gp.disansebastiano@unisalento.it (G.P.D.S.); 3 Studio Effemme-Chimica Applicata, Via Paolo VI, 73018 Squinzano (LE), Italy; stefano.mazzotta@studioeffemme.com

**Keywords:** physico-chemical properties, inorganic NPs, absorption, cytotoxicity, mosses

## Abstract

Noble metals nanoparticles (NPs) and metal oxide NPs are widely used in different fields of application and commercial products, exposing living organisms to their potential adverse effects. Recent evidences suggest their presence in the aquifers water and consequently in drinking water. In this work, we have carefully synthesized four types of NPs, namely, silver and gold NPs (Ag NPs and Au NPs) and silica and titanium dioxide NPs (SiO_2_ NPs and TiO_2_ NPs) having a similar size and negatively charged surfaces. The synthesis of Ag NPs and Au NPs was carried out by colloidal route using silver nitrate (AgNO_3_) and tetrachloroauric (III) acid (HAuCl_4_) while SiO_2_ NPs and TiO_2_ NPs were achieved by ternary microemulsion and sol-gel routes, respectively. Once the characterization of NPs was carried out in order to assess their physico-chemical properties, their impact on living cells was studied. We used the human colorectal adenocarcinoma cells (Caco-2), known as the best representative intestinal epithelial barrier model to understand the effects triggered by NPs through ingestion. Then, we moved to explore how water contamination caused by NPs can be lowered by the ability of three species of aquatic moss, namely, *Leptodictyum riparium*, *Vesicularia ferriei*, and *Taxiphyllum barbieri,* to absorb them. The experiments were conducted using two concentrations of NPs (100 μM and 500 Μm as metal content) and two time points (24 h and 48 h), showing a capture rate dependent on the moss species and NPs type. Then, the selected moss species, able to actively capture NPs, appear as a powerful tool capable to purify water from nanostructured materials, and then, to reduce the toxicity associated to the ingestion of contaminated drinking water.

## 1. Introduction

The widespread use of inorganic nanomaterials, namely, metal (Ag NPs and Au NPs) and metal oxide NPs (SiO_2_ NPs and TiO_2_ NPs) in a broad range of commercial products [1], such as paints [2], cosmetics [3], medicine [4,5], sensors [6,7], food additives [8,9,10], and sunscreens [11,12], highlighted the problem of their behavior and fate in several environmental compartments [13]. The unique physico-chemical properties, due to the high surface-to-volume ratio, make NPs very reactive materials with respect to the bulk counterpart [14,15]. However, their toxicological profile is still not fully understood.

The production and applications of nanomaterials, as well as the transformation in waste of different products containing them [16], lead to the release of NPs into all ecosystems including freshwater, marine water, soil, and atmosphere [17]. Therefore, nanomaterials can be found in the drinking water sources (rivers, lakes, reservoirs, or groundwater), and then in the food chain, undergoing some transformation processes such as degradation or agglomeration [17]. An indirect potential exposure route to NPs is the consumption of contaminated drinking water, triggering adverse effects especially in the gastrointestinal tract [18]. In fact, Ag NPs and Au NPs administered in mammals (rats and mice) by drinking water induced collateral outcomes strongly dependent on NPs physico-chemical properties such as size, shape, charge, and surface functionalization [19,20,21,22]. In general, the small size is more toxic than the larger one [23]. Furthermore, TiO_2_ NPs (classified as a group 2B, possibly carcinogenic substance for humans) caused strong toxicity in vivo, especially the anatase crystalline form [24,25,26] also low doses of SiO_2_ NPs [27] resulted to be harmful.

Taking this in mind, it is easy to understand the importance to reduce the concentration of NPs in water. The developed countries carefully specify the standards to follow for drinking water. For example, in Europe, the European Drinking Water Directive [28] establishes standards required by also the United States issued guidelines with the Safe Drinking Water Act drawn up by the United States Environmental Protection Agency (EPA) [29].

It is well known that the contamination of drinking water by NPs can occur by water treatment processes; for example, coagulation techniques are known to remove many kinds of contaminants, but their efficiency to extract NPs from water has not been clarified yet [30].

Some investigations have examined this topic in depth; in particular to ascertain TiO_2_ and/or Ag NPs removal during alum or ferric based coagulation [31,32,33]. Results showed that TiO_2_ NPs and Ag NPs present in a percentage ranging from 3% to 60% for TiO_2_ NPs and from 2% to 20% for Ag NPs. Other advanced drinking water treatments, namely, microfiltration, lime softening, and alum coagulation/activated carbon adsorption, reduced the percentage of Au NPs, Ag NPs, SiO_2_ NPs, and TiO_2_ NPs in drinking water [32].

However, a lot of disadvantages are related to these processes, in particular the high sludge production, and the consequent additional costs for its treatment since it is also toxic for humans. In addition, dissolved solid salts used in the treatment can alter the pH of water [34,35]. For these reasons, the research of new low-cost and nature-inspired techniques to remove NPs from water is necessary. In particular, the use of aquatic mosses can be a potential easy and biocompatible tool to absorb NPs from the water.

Mosses are non-vascular plants that absorb nutrients and pollutants from their entire vegetative biomass usually called gametophyte. Aquatic mosses, living submerged in water, maximize this absorbent capacity and because of their ramified structure, rich in leaflet-like structures, offer a large surface-to-weight ratio. Because of these characteristics and the clumped growth, mosses are a tridimensional matrix and very efficient for biofiltration [36]. Every moss showed specific morphological characteristics, including different abilities to perform the uptake of contaminants. The *Hypnaceae* family is the largest group of mosses; among them, some genera of aquatic mosses attract attention for their anatomical structure and their ability to uptake pollutants *Leptodictyum riparium* [37], *Vesicularia ferriei* [38], and *Taxiphyllum barbieri* [39].

*L. riparium* is a widespread species almost all over the world (except for Pacific islands and Australia) [40]. The interesting aspect of this species is that it has also been found in an acid mining lake, surviving at the level of volcanic craters up to a pH of 1.6 [41].

*V. ferriei*, commonly known as “Weeping moss”, is native to China and can be found in temperate East Asian regions of Japan and Philippines [42]. Under submerged conditions, it develops its characteristic overhanging growth habit and is densely ramified. This moss is relatively undemanding regarding lighting and nutritional needs.

*T. Barbieri* is a native species of Southeast Asia and it is also known as “Java moss”; it is the best-known moss belonging to the *Taxiphyllum* genus able to grow without difficulty in all types of water, including slightly brackish water. In addition, it lives under all types of light at temperature values ranging from 21 °C to 32 °C. 

The gametophyte of all three mosses grows as a complex reticulate mat, ideal to filter particles from water, but their ability to actively absorb ions may change and the uptake of NPs was not investigated with a comparative approach.

In this work, we investigated the ability of these mosses to filter-out different kinds of NPs, namely, Ag NPs, Au NPs, SiO_2_ NPs, and TiO_2_ NPs. We first synthesized NPs by different synthetic routes, to obtain spherical and monodispersed NPs with a negative charge surface and the same size (about 20 nm). Then, we tested the acute toxicity of these NPs at two doses (100 µM and 500 µM of metal concentrations) for 24 h and 48 h in colorectal cells (Caco-2) that are the best cellular model to assess the toxicity at the gastrointestinal level. Once we completed the evaluation of their toxicological profile, we used the three types of mosses to assess the absorption profile, exposing them to the NPs. The same concentrations used for cytotoxicity tests were applied in order to evaluate the most efficient moss to remove NPs from water. Then, the residual concentrations of NPs achieved after mosses experiments were used again to assess cell viability.

In our experiments, we demonstrated the possibility to use a specific moss to absorb a different kind of NPs. This experimental protocol could be suitable to remove nano contaminants from drinking water without the use of complex water treatment techniques. 

## 2. Materials and Methods

### 2.1. Reagents

Tetrachloroauric(III) acid (HAuCl_4_), silver nitrate (AgNO_3_), sodium citrate, tannic acid, nitric acid (HNO_3_), hydrochloric acid (HCl), Cyclohexane, Triton X-100, Tetraethyl orthosilicate 98% (TEOS) ammonium hydroxide 28.0–30.0% (NH_4_OH), titanium (IV) isopropoxide 99.9% (TTIP), DMEM (Dulbecco’s Modified Eagle’s Medium-high glucose), fetal bovine serum (FBS), penicillin-streptomycin, dimethyl sulfoxide (DMSO), glutaraldehyde, WST-8 assay, phosphate buffer saline (PBS), and bovine serum albumin (BSA) were purchased from Merck KGaA (Darmstadt, Germany).

Caco-2 cells were purchased from American Type Culture Collection (ATCC) (ATCC, Manassas, VA, USA). A total of 300 mesh amorphous carbon-coated Cu grids were purchased from Ted Pella Inc, USA. Petri dishes were purchased from corning (Corning, New York, NY, USA), and 4′,6-diamidino-2-phenylindole (DAPI) and phalloidin-FITC were purchased from Thermo Fisher (Waltham, MA, USA)

### 2.2. Synthesis of Ag NPs and Au NPs 

The colloidal syntheses of the Ag NPs and Au NPs were performed according to the procedure described in [43,44] which rely on the metal salt reduction in aqueous solution by using sodium citrate. For the Au NPs synthesis, a reaction flask, filled with 150 mL of HAuCl_4_ (0.25 mM) aqueous solution, was heated under reflux with a condenser and stirring, and then, 1.15 mL of 0.1 M sodium citrate was rapidly injected. The solution in the flask was kept at the boiling point until the color solution became red wine. For the synthesis of the Ag NPs, 1.5 mL of sodium citrate aqueous solution (1.36 mM) containing 2.9 μM of tannic acid was heated up to 60 °C. After the addition of AgNO_3_ (0.592 mM), the solution was heated under reflux with a condenser up to the boiling point until the color turned dark brown. The reaction solutions were then cooled down to room temperature and stored in the dark at 4 °C. After this step, the achieved NPs were centrifuged at 6000× *g* for 45 min and washed three times with Milli-Q water.

**Figure 1:**
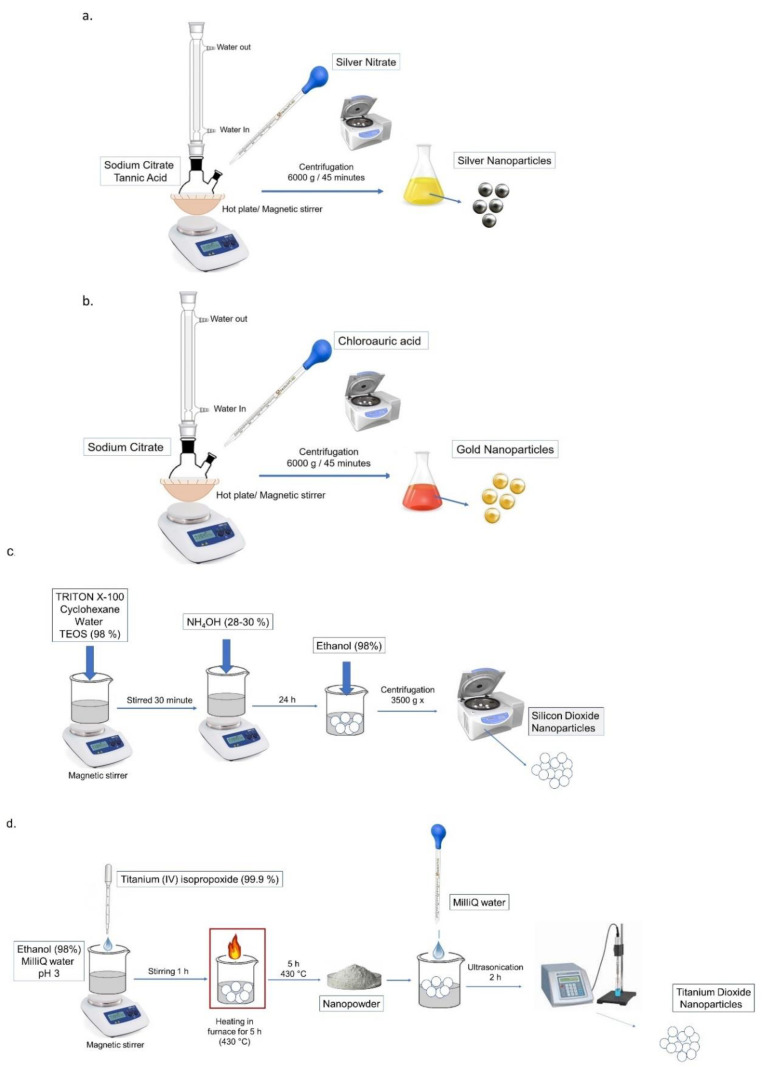
Procedure steps involved in the synthesis of Ag NPs (**a**), Au NPs (**b**) SiO_2_ NPs (**c**), and TiO_2_ NPs (**d**).

Schematic diagrams of the synthetic paths involved in the Ag NPs and Au NPs synthesis are shown in Figure 1a,b, respectively.

### 2.3. Synthesis of Amorphous SiO_2_ NPs and Crystalline TiO_2_ NPs

The ternary W/O microemulsion was prepared at room temperature by mixing water an organic solvent (cyclohexane), a surfactant (Triton X-100) following the methods described in [45]. Briefly, 880 μL of Triton X-100, 3.75 mL of cyclohexane, 170 mL of water, and 50 μL of TEOS (98%) were mixed and stirred for 30 min. Later, 30 μL of NH_4_OH (28.0–30.0%) was added to the microemulsion. After 24 h, the suspension was separated by centrifugation (3500× *g*) followed by five washes in ethanol (98%), and Milli-Q water; finally, the NPs were dispersed in water.

TiO_2_ NPs were prepared following the sol-gel method described by Leena et al. [46] with some modifications. Briefly, TTIP (99.9%) was dropped in a solution of ethanol and Milli-Q water with a molar ratio of 5:1:1 under stirring in acidic conditions (pH 3) for 1 h. NPs were incubated for 5 h at 30 °C followed by heating at 430 °C for 3 h to obtain a white nano powder. Lastly, the NPs were dispersed in water (st 0.2%) and ultrasonicated for 2 h in order to reduce the aggregation and enhance stability. 

The schematic representations of the synthetic paths involved in the Ag NPs, Au NPs, SiO_2_ NPs, and TiO_2_ NPs syntheses are shown in Figure 1a–d, respectively.

### 2.4. Inductively Coupled Plasma Emission Spectroscopy (ICP-OES)

The concentrations of the Ag NPs, Au NPs, SiO_2_ NPs, and TiO_2_ NPs were estimated by elemental analyses performed by using ICP-OES Perkin Elmer AVIO 500. A total of 250 µL of the Ag NPs, SiO_2_ NPs, and TiO_2_ NPs were digested, overnight, by adding 2 mL of HNO_3_, whereas for Au NPs solutions, 2 mL of aqua regia, was used. After this step, the solutions were diluted with Milli-Q water (1:5) before the measurements. 

### 2.5. Transmission Electron Microscope (TEM) Measurements, Dynamic Light Scattering (DLS), ζ-Potential Measurements, UV–vis Analysis, X-ray Diffraction (XRD) Measurements

TEM images were recorded by JEOL JEM 1011 microscope operating at an accelerating voltage of 100 kV. TEM samples were prepared by dropping a dilute solution of NPs in water on carbon-coated copper grids (Formvar/Carbon 300 Mesh Cu). Size statistical analysis of the NPs size distribution was performed by using the ImageJ software and the results were fitted by the OriginPro software (OriginLab Corporation, Northampton, MA, USA). 

DLS and ζ-potential measurements were performed by a Zetasizer Nano ZS90 (Malvern, PA, USA) equipped with a 4.0 mW HeNe laser operating at 633 nm and an avalanche photodiode detector. Acquisitions were performed at 25 °C in an aqueous solution at pH 7. The optical absorbance profile of Ag NPs, Au NPs, SiO_2_ NPs, and TiO_2_ NPs aqueous solutions were measured with a Cary 300 UV–vis spectrophotometer (Varian, Palo Alto, CA, USA) at a resolution of 1 nm using a 5-mm path length quartz cuvette. Powder X-ray diffraction (XRD) for Au NPs, Ag NPs, SiO_2_ NPs, and TiO_2_ NPs was performed on a Rigaku, diffractometer in Bragg–Brentano reflection geometry using filtered Cu-Ka radiation. The XRD patterns were recorded in the range of 2Q ¼ 20–80 by step scanning, using 2Q increments of 0.02 and a fixed counting time of 2 s/step.

### 2.6. Cell Culture

Caco-2 (ATCC^®^ HTB-37™) were maintained in DMEM with 50 μM glutamine, supplemented with 100 U/mL penicillin/streptomycin 100 mg/mL and 20% of FBS. Cells were incubated in a humidified controlled atmosphere with a 95% to 5% ratio of air/CO_2_, at 37 °C.

### 2.7. Viability Assay 

5 × 10^3^ Caco-2 cells/well were seeded in 96 well microplates. After 24 h of stabilization, cells were exposed to 100 µM to 500 µM of Ag NPs, Au NPs, SiO_2_ NPs, and TiO_2_ NPs for 24 h and 48 h. DMSO was used as a positive control. At the endpoints, cell viability was investigated by using a standard WST-8 assay following the procedure previously described [5]. Differences in cell viability between cells treated with NPs and the control were statistically significant performing a Student’s *t*-test with a *p*-value of <0.05 (<0.05 *). 

### 2.8. Confocal Analysis

Caco-2 cells were seeded at a concentration of 7 × 10^4^ cells/mL in glass Petri dishes. After 24 h of stabilization, the culture media was removed and replaced with Ag NPs, Au NPs, SiO_2_ NPs, and TiO_2_ NPs solutions at a concentration of 500 µM for each one. After 48 h of incubation, NPs solutions were removed, and cells were washed with Phosphate Buffered Saline (PBS).

Samples were fixed by using glutaraldehyde (0.25%) for 10 min, and then cells were permeabilized by Triton X-100 (0.1%) for 5 min. Nuclei were labeled with 1 μg/mL of DAPI (5 min), whereas F-actin was stained using 1 µg/mL of phalloidin-FITC for 1 h. Acquisitions were performed by Zeiss LSM700 (Zeiss, Oberkochen, Germany) confocal laser scanning mounted on an Axio Observer Z1 (Zeiss, Germany) inverted microscope, using the Alpha Plan-Apochromat (Zeiss, Germany) 100× oil-immersion objective with 1.46 NA. Coherency values of F-actin were performed on 20 cells, using the OrientationJ plugin of the ImageJ 1.47 software. This parameter describes the orientation degree of actin fibers: more disordered fibers have values near 0, whereas aligned ones show a coherency value of about 1. The coherency parameter was measured choosing a specific ROIs in confocal acquisitions, based on the measure of the structure tensors in a local neighborhood. Simultaneously, the software calculated the value of orientation and coherency. 

### 2.9. Plant Material

Aquatic mosses belonging to three different species were grown in polypropylene Steri Vent containers (107 × 94 × 96 mm) submerged with fresh water with no supplements and incubated in controlled growing chambers, to reduce external contaminants, at 22 °C and 150 µmol/m^2^ sec light intensity for a 16 h light period. *Taxiphyllum barbieri* and *Vesicularia ferriei* were obtained from commercial source (Tropica Aquarium Plants; Mejlbyvej 200 8250 Egå, Denmark) while *Leptodictyum riparium* derived from the Botanical Garden of the University of Naples “Federico II,” Italy, and used in several previous studies [37,47]. Young gametophyte fragments were sampled from in vitro cultivated mats, and carefully cleaned and washed with deionized water before the experimental treatment.

### 2.10. Plant Treatment with NPs

**Figure 2:**
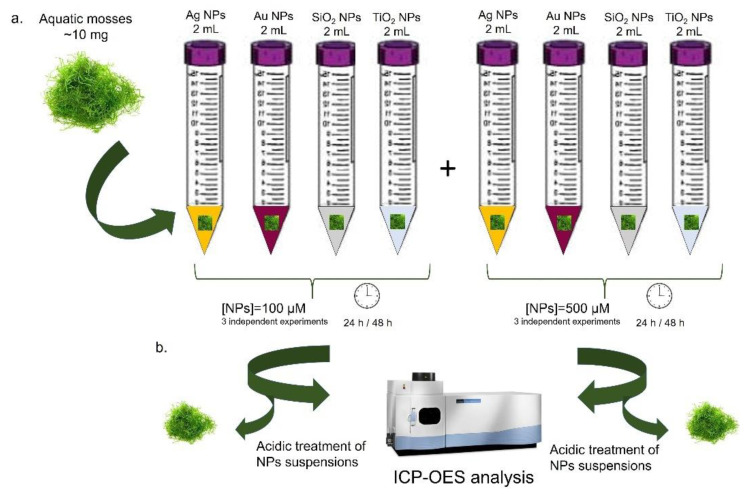
Schematic representation of aquatic mosses treatments with different kinds of NPs at two concentrations (100 μM and 500 μM) for 24 h and 48 h (**a**). After incubation, mosses were removed, and the residual NPs solutions were analyzed by ICP-OES (**b**). The detailed procedure was described in the 2.10 paragraph.

Excess of water was removed from 10 mg mosses (*Taxiphyllum barbieri*, *Vesicularia ferriei*, and *Leptodictyum riparium*) gametophyte explants by blotting on filter paper before transfer in 15 mL test-tubes containing 2 mL of freshwater NPs suspension at room temperature. Four kinds of NPs were tested: Ag NPs, Au NPs, SiO_2_ NPs, and TiO_2_ NPs. Each was applied at two different concentrations, 100 µM and 500 µM. The mosses were incubated with the NPs suspensions for 24 and 48 h at 25 °C to test the ability to subtract NPs from the suspensions. At the end of the incubation, mosses were separated from water containing NPs. A total of 1 mL of HNO_3_ was added to solutions containing Ag NPs, SiO_2_ NPs, and TiO_2_ NPs. Moreover, 1 mL of Aqua Regia (HCl: HNO_3_, 3:1) was added to Au NPs solutions. Later, the samples were diluted with Milli-Q water in a ratio of 1:10 and analyzed by ICP-OES to the final volume of 10 mL. In the experimental protocol, three replicates for each NPs type and concentration were used. A schematic diagram regarding the plant treatment with NPs is shown in Figure 2. 

## 3. Results and Discussion

**Figure 3:**
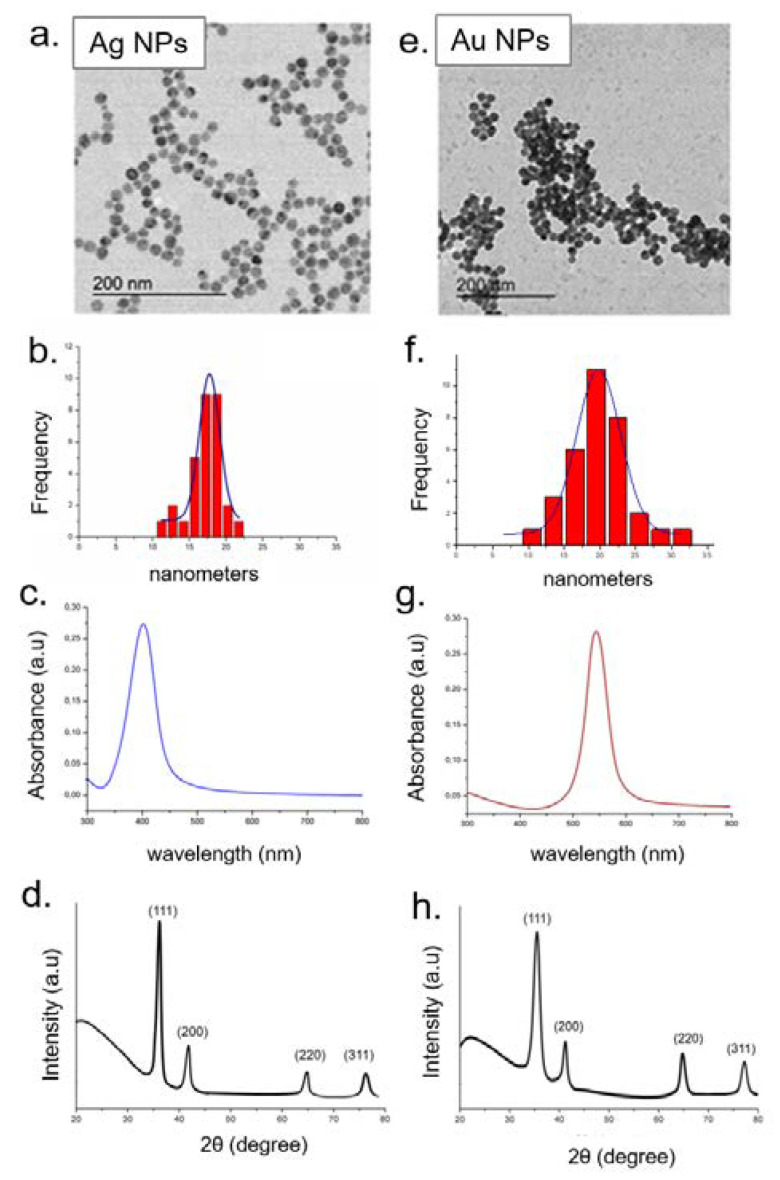
Representative TEM images of Ag NPs (**a**) and Au NPs (**e**). Statistical analysis with Gaussian fit (black line) (**b**–**f**), UV–vis (**c**–**g**), and XRD spectra (**d**–**h**) of Ag NPs and Au NPs, respectively.

Ag NPs and Au NPs were synthetized by a colloidal route using metal salts (AgNO_3_ and HAuCl_4_) and citrate sodium salt as reducing agents. The synthetic procedure was conducted at high temperature under flux, permitting to obtain monodispersed and spherical NPs with a size of about 20 nm, as demonstrated by TEM acquisitions (Figure 3a,e). The statistical analysis was performed on 70 of each metal NPs type by the ImageJ software tool (Particle Analysis) in order to record the average diameter, which was (18 ± 3) nm for Ag NPs and (20 ± 2) nm for Au NPs (Figure 3b,f). The monodispersion of metallic NPs was also confirmed by UV–vis measurements. Both Ag NPs and Au NPs displayed sharp plasmon peaks recorded to 400 nm and 550 nm, respectively, that are typical spectra of monodispersed spherical metallic NPs (Figure 3c–g). 

The XRD profile of Ag NPs and Au NPs is shown in Figure 3d,h, respectively. The XRD pattern of Ag NPs exhibited characteristic peaks indexed to the (111), (200), (220), (311) Bragg’s reflections of a face-centered cubic (FCC) structure of metallic Ag. The correspondent scattering angles (2θ) were 38.4°, 44.6°, 64.5°, 77.7° indicating that Ag NPs were pure crystalline as the values were correspondent to the standard for Ag collected of the Joint Committee on Powder Diffraction Standards (JCPDS) (Figure 3d). Furthermore, in the case of Au NPs, we observed the characteristic diffraction peaks of metallic gold phase at 38.21°, 44.39°, 64.62°, and 77.59° correspondent to the standard for Au collected of the JCPDS (Figure 3h).

**Figure 4:**
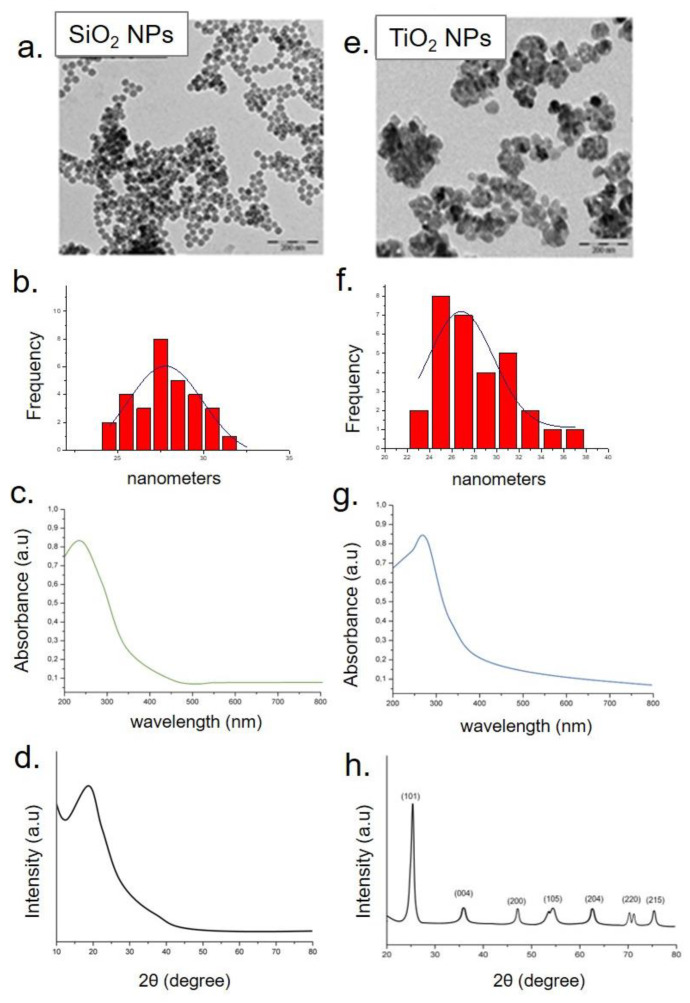
Representative TEM images of SiO_2_ NPs (**a**) and TiO_2_ NPs (**e**). Statistical analysis with Gaussian fit (black line) (**b**–**f**), UV–vis (**c**–**g**), and XRD spectra of SiO_2_ NPs and TiO_2_ NPs, respectively (**d**–**h**).

The same characterizations were performed on amorphous SiO_2_ NPs and crystalline TiO_2_ NPs. The TEM images showed two different morphologies for both types of metal oxide NPs. In particular, SiO_2_ NPs appeared spherical and monodispersed, whereas the TiO_2_ NPs were irregular in morphology (Figure 4a,e). The statistical analysis performed on TEM acquisitions revealed the accurate size of NPs that was (23 ± 4) nm for SiO_2_ NPs and (25 ± 6) nm for TiO_2_ NPs (Figure 4b–f). The UV-Vis analysis displayed typical peaks at around 260 nm and 295 nm for SiO_2_ NPs and TiO_2_ NPs (Figure 4c–g).

The analysis of the XRD pattern did not show sharp Bragg diffraction peaks that confirm the amorphous nature of SiO_2_ NPs (Figure 4d). Contrarily, the dominant peaks at 2θ  =  25.4° (101), 37.89° (A004) 48.1° (200), 54.005° (105), 62.4° (204), 70.4° (220), and 75.2 °C (215) were distinctive of the TiO_2_ anatase phase, confirming their crystalline nature. The peaks match the standard JCPDS data well (Figure 4h).

DLS measurements corroborated the TEM data (Table 1), observing hydrodynamic diameters of (20 ± 3) nm and (19 ± 2) nm for the Ag NPs and Au NPs, respectively. Similar surface charges, (−33 ± 2) mV and (−27 ± 3) mV, were also quantified for Ag NPs and Au NPs, respectively, due to the citrate capping formation. Conversely, the DLS measurements of SiO_2_ NPs and TiO_2_ NPs showed a diameter of (23 ± 4) nm and (22 ± 5) nm, respectively. In addition, their surface charge was (−25 ± 4) mV for SiO_2_ NPs and (−23 ± 2) mV for TiO_2_ NPs (Table 1).

After one month, the stability of the four types of NPs was carried out by the size and charge measurements comparing them with fresh prepared NPs. The results obtained did not show any significant change in NPs parameters reflecting the high stability of the produced NPs (Table 2). 

**Figure 5:**
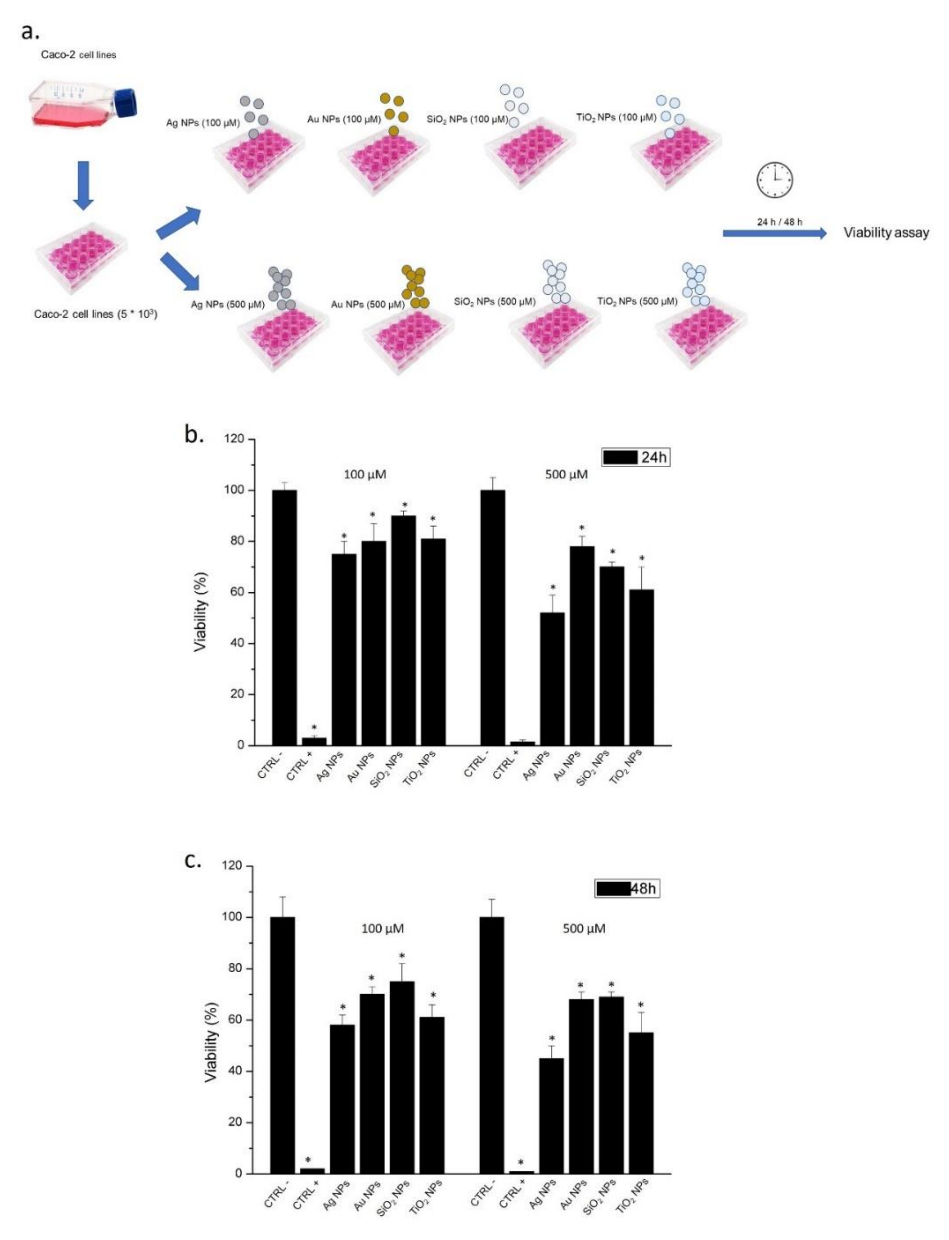
Schematic representation of cells treatment steps by the use of different kinds of NPs as described in materials section (**a**); viability (WST-8) assay of Caco-2 cells after 24 h and 48 h of exposure to Ag NPs, Au NPs, SiO_2_ NPs, and TiO_2_ NPs (**b**,**c**). The viability of NPs-treated cells was normalized to non-treated control cells (CTRL-). As positive control (P), cells were incubated with 5% Dimethyl Sulfoxide (DMSO) (CTRL+). Data reported as mean ± SD from three independent experiments are considered statistically significant compared with control (*n* = 8) for a *p-*value of ˂0.05 (<0.05 *).

Once the NPs characterization was completed, we used NPs to test their potential toxicity in Caco-2 cells, which is the best model to assess the alterations of the gastrointestinal tract. The aim of this test was to mimic the potential impact of NPs assimilated by contaminated drinking water. We used two concentrations of NPs, 100 μM and 500 μM for 24 h and 48 h (Figure 5a), showing a reduction of viability that was time and doses dependent. In particular, the Ag NPs at the higher concentration tested, induced a viability reduction of about 50% after 24 h, whereas the treatment with Au NPs and SiO_2_ NPs caused softer effects; in fact, the reduction of viability was about 20% and 30%, respectively. TiO_2_ NPs triggered a viability decrease of 40% (Figure 5b). After 48 h, the lethal effects became more evident, but altogether, they maintained a trend similar to the 24 h (Figure 5c).

**Figure 6:**
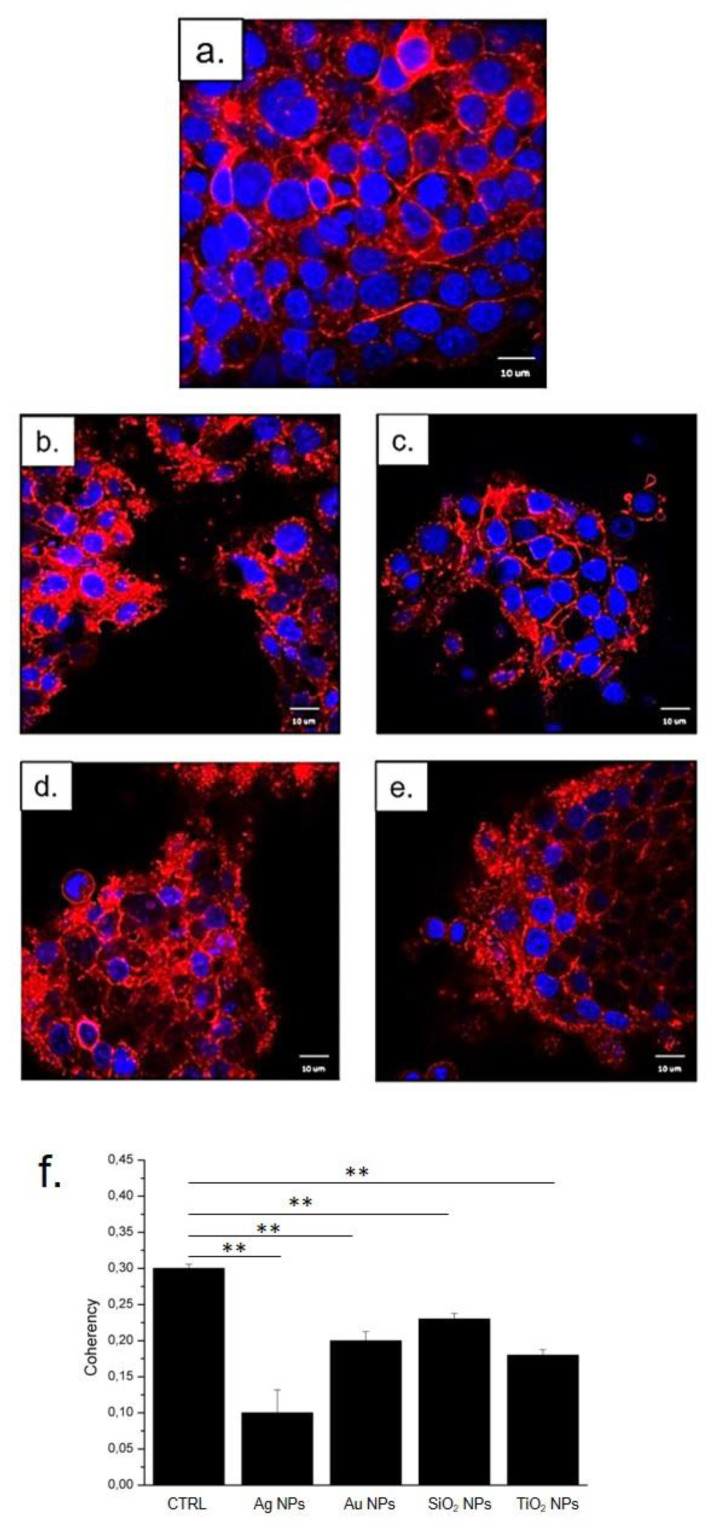
Effects of Ag NPs, Au NPs, SiO_2_ NPS, and TiO_2_ NPs on the actin network of Caco-2 cells treated with 500 µM of NPs for 48 h: (**a**) control, (**b**) Ag NPs, (**c**) Au NPs, (**d**) SiO_2_ NPs, (**e**) TiO_2_ NPs. Cells were fixed and then stained with Phalloidin–ATTO 488 and DAPI. The 2D images of cortical actin were acquired by a Zeiss LSM700 (Zeiss) confocal microscope equipped with an Axio Observer Z1 (Zeiss) inverted microscope using a ×100, 1.46 numerical aperture oil immersion lens. All data were processed by the ZEN software (Zeiss). (**f**) Coherency values were expressed as a mean value and relative SD, calculated from confocal acquisitions by ImageJ (calculation on 15 cells). The mean values and their standard deviations were reported in the histograms. Results were statistically significant for *p* < 0.01 (< 0.01 **).

In order to confirm the data obtained with the MTT test, we performed confocal analysis to visualize the possible damage to the actin morphology. The actin fibers are important to maintain the cell shape and physiological behavior. When we incubated Caco-2 cells, with NPs (500 μM) for 24 h, we observed a substantial modification of the cytoskeleton with respect to the control (untreated cells). As shown in Figure 6b–e after incubation with the four types of NPs, we observed a perturbation of the actin network. The actin fibers appeared disorganized and dissolved, and cells tended to lose their connections compared with control cells (Figure 6a). This behavior was noticeable when cells were exposed to Ag NPs (Figure 6b), Au NPs (Figure 6c), SiO_2_ NPs (Figure 6d), and TiO_2_ NPs (Figure 6e). However, in good agreement with the results obtained with the viability test, the stronger effects became more evident in cells treated with Ag NPs (Figure 6b). In order to complete the imaging studies, coherency analysis was adopted by using the ImageJ software. This parameter gives information on the degree of fiber orientation compared to the surroundings. Untreated Caco-2 cells showed a coherency value of 0.3 ±  0.04; after exposure to NPs, the value decreased. Ag NPs induced the stronger coherency value reduction (0.08 ±  0.02) with respect to the other types of NPs studied. Indeed, the Au NPs triggered the reduction to 0.22 ± 0.05, whereas the values related to SiO_2_ NPs and TiO_2_ NPs were 0.24 ± 0.02 and 0.18 ±  0.03, respectively. These results validated the actin disorganization observed in confocal acquisitions, underlining the toxic behavior of the four kinds of NPs.

Therefore, the importance to reduce the NPs amount in drinking water is a crucial challenge for the future due to the large use of nanomaterials in a great number of industrial processes and commercial products. In particular, it is preferred to use green alternatives to impact the quality of water as little as possible and making the further contamination that can occur using the conventional water treatments difficult. From here comes the idea to use three species of aquatic mosses to assess their ability to adsorb the different kind of synthesized NPs. 

**Figure 7:**
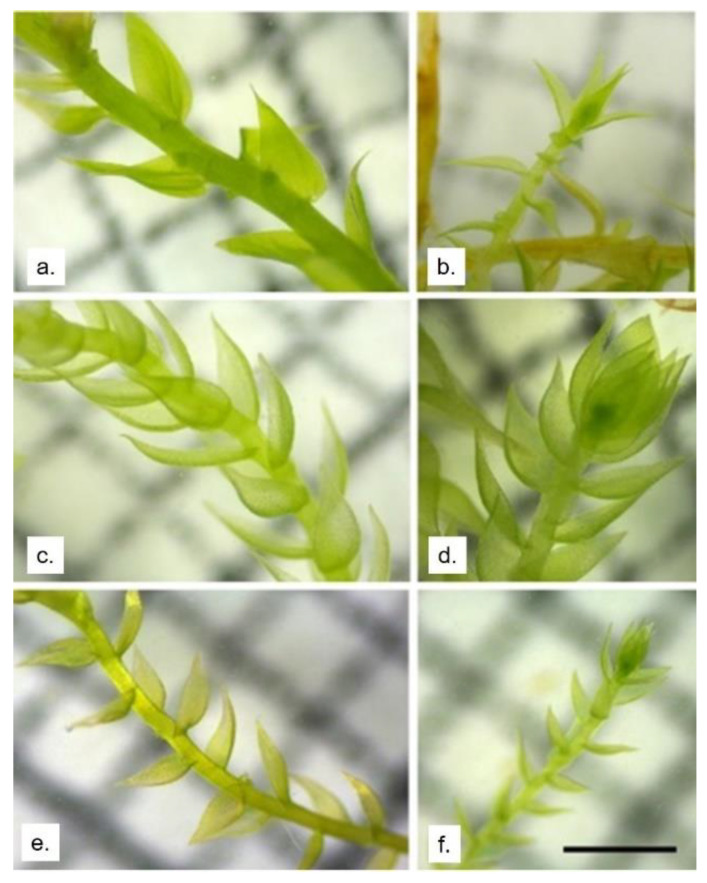
Stereoscopic images of the gametophyte of the three mosses. (**a**) Stem and leaflets of *L. riparium*; (**b**) apical growth in *L. riparium*; (**c**) stem and leaflets of *V. ferriei*; (**d**) apical growth in *V. ferriei*; (**e**) stem and leaflets of *T. barbieri*; (**f**) apical growth in *T. barbieri*. Scale bar 1 mm.

The aquatic mosses used were very similar but morphologically distinguishable. *L. riparium* has stem leaves egg-to-spearhead-shaped, widest just above the base, and tapering evenly to the narrowly pointed tip. It can help to identify this moss, the widely spreading, often widely spaced leaves, and the shape of the leaf base, which is narrowed at the junction with the stem (Figure 7a). The shoots often appear flattened, but they are variable, and some forms have curved leaves. Branch leaves are similar to stem leaves but smaller [47]. The apical growth of the gametophyte has few leaves, and these are flatter and narrower than the stem leaves (Figure 7b). *V. ferriei* have shortly spaced ovate dorsal leaves and suborbicular lateral leaves with shortleaf apices, which are broadly acute or obtuse [42]. The leaf base is a little bit narrower than the center of the leaf (Figure 7c). The leaves in the shoot apex are very close (Figure 7d).

*T. barbieri* has flattened leaves arranged on two sides of the stem and branches. The leaf shape is oval oblong with a short apex [48,49] and the leaf base is not particularly narrower (Figure 7e). Moreover, the apical leaves of the stem are few, flattened, and smaller (Figure 7f). Their ability to trap NPs may change not just for their morphology but also for their physiologic ability to actively internalize them.

The three mosses were exposed to the four synthesized NPs. Firstly, we prepared NPs water solutions at two concentrations (100 μM and 500 μM) for each NPs type. The correspondent values expressed as mass/volume (mg/mL) are reported in Table 3.

The NPs were completely dissolved in water without visible decantation phenomena. Then, 10 mg of gametophytes from three mosses were collected and immersed in 2 mL of NPs water solutions (100 μM and 500 μM). Three replicas were prepared per experiment. After 24 and 48 h, the gametophytes were moved away, and the solutions were analyzed with ICP-OES to obtain the residual concentrations of NPs after the exposure to the mosses.

**Figure 8:**
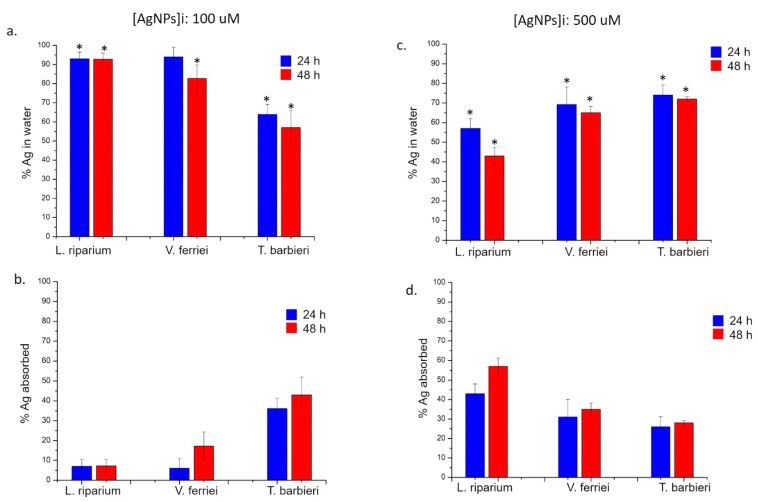
ICP-OES measurements performed after the exposure of *L. riparium*, *V. ferriei*, and *T. barbieri* to 100 μM and 500 μM of Ag NPs. (**a**,**c**) Percentage of silver in water was obtained after plant remotion (24 h and 48 h) and further ICP-OES analysis. (**b**,**d**) Percentage of silver absorbed by the mosses; the values were calculated by difference of initial the concentrations (100 μM and 500 μM) and final concentrations found after 24 h and 48 h of plants exposure. Data reported are mean ± SD from three independent experiments and they are considered statistically significant compared with control represented by Milli-Q water (data not shown) for a *p*-value of ˂0.05 (<0.05 *).

Among the three aquatic plants, a different trend of absorption was observed; the reduction of metallic NPs and metal oxide NPs concentration was evidently influenced by the plant types. In general, the absorption rate was time and dose dependent. In detail, observing the data obtained for the Ag NPs, it was evident that the *T. barbieri* was able to remove the NPs from water at a concentration of 100 μM much more than the other two species both at 24 and 48 h. Sure enough, the absorbed percentage of Ag NPs turned out to be about 38% after only 24 h, and up to 43% after 48 h (Figure 8a,b). Using the concentration of 500 μM, it was observed instead that the most suitable species to retain this concentration turned out to be *L. riparium* especially after 48 h, retaining a percentage of about 55% (Figure 8c,d). These observations pointed out a different behavior due to the specific moss biology, better than to morphology.

However, all species had the ability to filter Ag NPs, albeit with less efficiency than the *L. riparium*. After 48 h, *V. ferriei* retained approximately 35% of NPs, while *T. barbieri* retained 30%.

**Figure 9:**
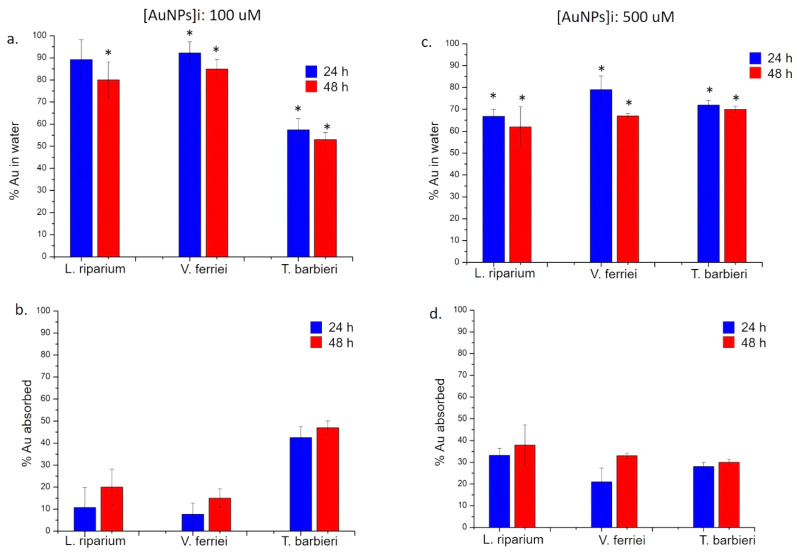
ICP-OES measurements performed after the exposure of *L. riparium*, *V. ferriei*, and *T. barbieri* to 100 μM and 500 μM of Au NPs. (**a**,**c**) Percentage of Au in water was obtained after plants remotion (24 h and 48 h) and further ICP-AES analysis. (**b**,**d**) Percentage of Au absorbed by Table (100 μM and 500 μM) and the final concentrations found after 24 h and 48 h of plant exposure. Data reported are mean ± SD from three independent experiments and they are considered statistically significant compared with control represented by Milli-Q water (data not shown) for a *p*-value of ˂0.05 (<0.05 *).

The absorption profile related to Au NPs was similar to the Ag NPs (Figure 9); as a matter of fact, after exposure to 100 μM of Au NPs, the *T. barbieri* resulted more suitable to absorb Au. After 24 h, this moss was able to filter about 42% of NPs, while at 48 h the percentage reached 44%. Moreover, in this case, the other two species were less efficient in the absorption process (Figure 9a,b).

At the highest concentration of Au NPs, a similar behavior found for Ag NPs was detected. In particular, the *L. riparium* was able to absorb about 40% of Au after 48 h, while values of 34% and 30% were measured in *V. ferriei* and *T. Barbieri*, respectively (Figure 9c,d).

**Figure 10:**
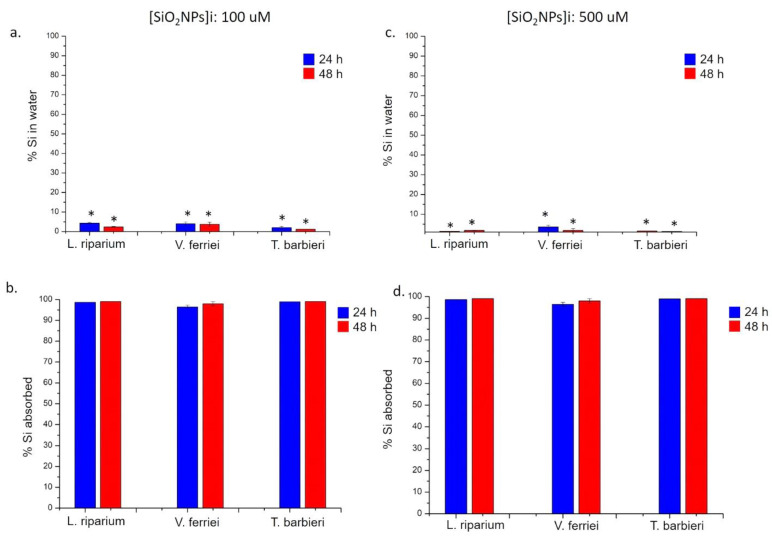
ICP-OES measurements performed after the exposure of *L. riparium*, *V. ferriei*, and *T. barbieri* to 100 μM and 500 μM of SiO_2_ NPs. (**a**,**c**) Percentage of Si in water was obtained after plants remotion (24 h and 48 h) and further ICP-AES analysis. (**b**,**d**) Percentage of Si absorbed by the mosses; the values were calculated by the difference of initial concentrations (100 μM and 500 μM) and the final concentrations found after 24 h and 48 h of plant exposure. Data reported are mean ± SD from three independent experiments and they are considered statistically significant compared with control represented by Milli-Q water (data not shown) for a *p*-value of ˂0.05 (<0.05 *).

**Figure 11:**
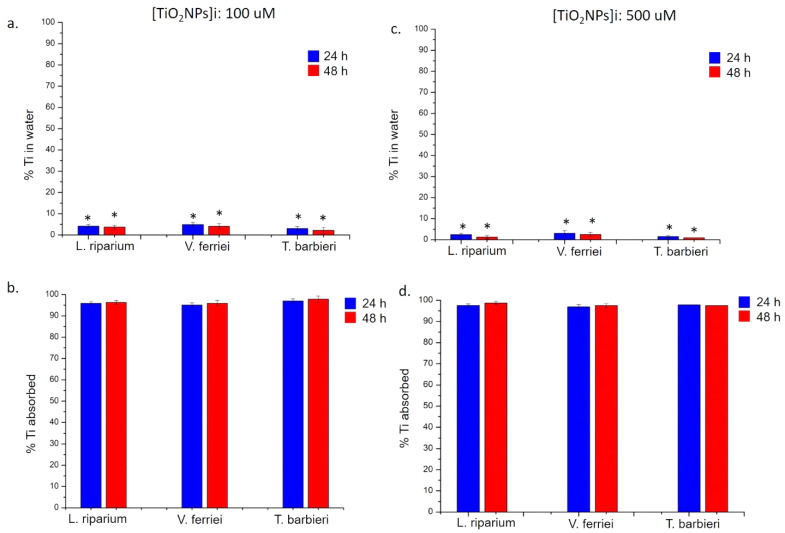
ICP-OES measurements performed after the exposure of *L. riparium*, *V. ferriei*, and *T. barbieri* to 100 μM and 500 μM of TiO_2_ NPs. (**a**,**c**) Percentage of Ti in water was obtained after plants remotion (24 h and 48 h) and further ICP-AES analysis. (**b**,**d**) Percentage of Ti absorbed by the mosses; the values were calculated by the difference of initial concentrations (100 μM and 500 μM) and the final concentrations found after 24 h and 48 h of plant exposure. Data reported are mean ± SD from three independent experiments and they are considered statistically significant compared with control represented by Milli-Q water (data not shown) for a *p*-value of ˂0.05 (<0.05 *).

The absorption analysis of the three mosses related to SiO_2_ NPs and TiO_2_ NPs showed a surprisingly different profile compared to noble metals NPs (i.e., Au NPs and Ag NPs). It was demonstrated that the three mosses were able to remove from solution both SiO_2_ NPs (Figure 10) and TiO_2_ NPs (Figure 11) with an efficiency of >95% after 24 h and 48 h using the two concentrations. Then, there was a very high difference between the absorption of these NPs compared to the others used in this study (Ag NPs and Au NPs).

The data obtained confirmed the idea to apply the mosses to remove NPs contamination from the water, because the initial concentrations of NPs tested (100 and 500 μM) were higher than the measured or predicted concentrations in environment and hydric basins [50]. For instance, it was estimated that the concentration of TiO_2_ NPs in water environment approximately ranged from 3 ng/L to 10 µg/L [51,52]. The value regarding SiO_2_ NPs was around to 0.7 ng/L, whereas Ag NPs and AuNPs showed higher concentration range values that were 10–100 ng/L and 140 ng/L [53,54,55]. Then, in the light of these data, our experiments demonstrated that the analyzed mosses had a great ability to absorb high NPs concentrations, typically present even in wastewaters. Moreover, the characterization of the four type of NPs was conducted after the mosses absorption to evaluate if the plants produced some kind of NPs aggregation or alterations (Table 4). The DLS and zeta potential of residual NPs concentrations after 48 h starting from the 500 μM of concentration demonstrated that the size and surface charge substantially remained unchanged. Only TiO_2_ NPs showed a greater tendency to aggregation which, however, has been previously demonstrated (Table 2) by placing them in water just after synthesis and leaving them for a month. 

The results obtained from ICP analysis highlighted that *T. barbieri* was more efficient to hold Ag NPs and Au NPs at the lowest initial concentration (100 μM), while when the concentration was 500 μM, *L. liparium* was more able to retain NP_S_. In the case of SiO_2_ NPs and TiO_2_ NPs, all three mosses had a very high efficacy in absorbing.

Then, in order to understand if a toxicity reduction can be achieved, we carried out a viability test on Caco-2 cells, incubating them with the residual NPs concentration found after the absorption of *T. barbieri* and *L riparium* for 48 h. Starting from the initial concentration of 100 μM, the measured residual concentration of AgNPs after exposure with *T barbieri* was 57 μM, whereas after exposure to 500 μM of concentration, the residual concentration value was 215 μM due to the *L. riparium* absorption.

The values become 53 μM and 310 μM for *T. barbieri* and *L.liparium* exposed to Au NPs, respectively. Concerning TiO_2_ NPs and SiO_2_ NPs, the concentrations used were 2.5 μM and 1.8 μM that correspond to the residual concentration after the same mosses described for Ag NPs and Au NPs.

**Figure 12:**
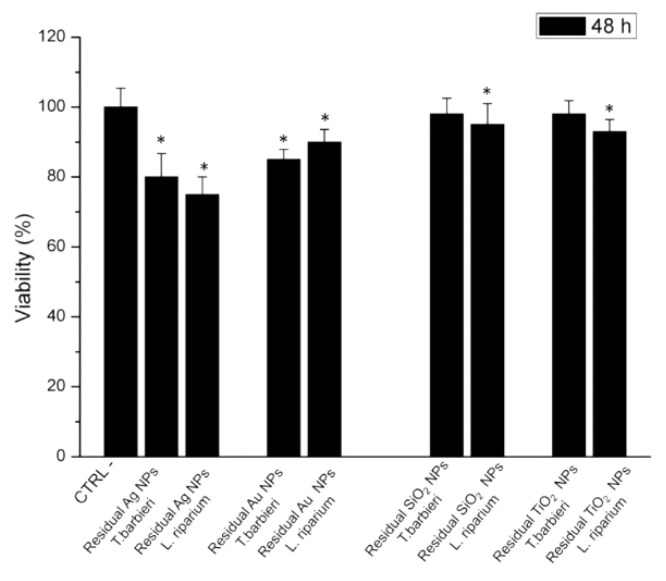
Viability (WST-8) assay of Caco-2 cells after 24 h and 48 h of exposure to residual concentration of Ag NPs, Au NPs, SiO_2_ NPs, and TiO_2_ NPs measured after the absorption induced by *T. barbieri* and *L. riparium* for 48 h. The viability of NPs-treated cells was normalized to non-treated control cells (CTRL-). As positive control (P), cells were incubated with 5% Dimethyl Sulfoxide (DMSO) reported a 98% of cell dead (data not shown). Data reported as mean ± SD from three independent experiments are considered statistically significant compared with control (*n* = 8) for a *p-*value of ˂0.05 (<0.05 *).

As displayed in histogram reported in Figure 12 the toxicity of the NPs to Caco-2 cells was very low compared to the same investigated using the initial concentrations of 100 μM and 500 μM. These interesting results demonstrated that this strategy can be useful and innovative to retain NPs and purify drinking water. 

The behavior of absorption could be due to different parameters similar to those used for the algae [56] in which the pseudo-second order kinetic model and Langmuir isotherm model were demonstrated [57,58] to understand the adsorption kinetic profile. Then, when the initial concentration of NPs increased, the absorption phenomena is also better until the ability to uptake NPs decreased. In addition, the interaction of the cell walls with the NPs was important in this interaction phenomenon. As previously demonstrated for the algae [59,60], some wall component, such as cellulose, contains glycoproteins and polysaccharides that can act as binding sites to induce the adsorption of NPs by mosses. In fact, the hydroxyl groups exposing SiO_2_ NPs and TiO_2_ NPs could be responsible for a deeper and stronger interaction with the gametophyte surface. Since the cell wall is a highly porous and complex molecular structure, we may be still far from the saturation of all molecular interactions.

Cellulose microfibrils carry many hydroxyl groups available for reactions. However, the main potential of living tissues is the presence of an extremely heterogeneous variety of molecules including enzymes.

In the case of Ag NPs and Au NPs having a capping of citrate, the interaction may be less specific. Citrate capping could induce a steric hindrance, that reduces the absorption efficiency on the gametophyte surface. Therefore, the physico-chemical properties of the NPs strongly influence the filtration. Since they had the same size and surface charge, the difference could be due to the type of chemical bonds that are established on the surface of the gametophytes or to an active uptake mechanism by the cell, for example, by endocytosis.

The cell wall by itself, given to the cellulose and pectins, can be used as a pollutant adsorbent and it was previously shown that also dry moss biomass can be a valid biosorbent [37,61].

Nonetheless, the physiological activity of mosses also contributes to the adsorption. A recent study [37] showed that the uptake of some specific pollutants, cationic lead, and chromium, in particular, is strongly influenced by the physiology of the moss. By washing weakly bound elements and removing pectins completely, it was shown that cellular elements different from cell wall play a big role. If the physiologic state of the same moss species is important, different species may offer an extremely diversified panel of opportunities that we just start to explore.

## 4. Conclusions

The growing consumption of commercial products containing nanomaterials could be a serious problem, due to their ability to reach water basins, many of which are used as a source of water for civil consumption. Wastewater treatment routes could also produce nanostructured contaminants. In this experimental work, we tested a potential biocompatible and eco-friendly alternative to the removal of contaminants in drinking water. This is possible thanks to the use of three different aquatic mosses that have been tested for their ability to absorb four different kinds of NPs (Ag NPs, Au NPs, SiO_2_ NPs, and TiO_2_ NPs) synthesized with likewise different techniques. These NPs have been shown to be toxic to the intestinal epithelial barrier model, which is the most exposed target to NPs contained in drinking water. Our experiments demonstrated that aquatic moss can be used for the removal of metallic NPs. Biodiversity among these organisms may assure the selection of optimal bio-filters accordingly to the contamination problem. *T. barbieri* was most effective absorbing metallic NPs, namely, Ag and Au, in a short period of time, but *L. riparium* showed the best performance in the time aspect. This evidenced that while *T. barbieri* superiority resides on morphology and cell wall characteristics, *L. riparium* superiority resides on long-term uptake processes. All three plants were capable to absorb SiO_2_ NPs and TiO_2_ NPs with an efficiency greater than 95% and further investigations are needed to distinguish between adsorption and relative mechanisms in different species. Interestingly, the reduction of NPs concentration in water makes it able for mosses to reduce cell toxicity of about 70–80% for Ag NPs and Au NPs and about 95% as demonstrated by viability tests conducted on residual NPs concentration after moss experiments.

Anyhow, these mosses could be used within polluted water reservoirs to filter water intended for civil use without using expensive and energy-consuming methods with a heavier carbon footprint.

## Figures and Tables

**Table 1:** Characterization of Ag NPs, Au NPs, SiO_2_ NPs, and TiO_2_ NPs in water by DLS and ζ-potential (mV) measurements.

Samples	Size (nm)	ζ-Potential (mV)
Au NPs	20 ± 3	−33 ± 2
Ag NPs	19 ± 2	−27 ± 3
SiO_2_ NPs	23 ± 4	−25 ± 4
TiO_2_ NPs	22 ± 5	−23 ± 2

**Table 2:** Characterization of Ag NPs, Au NPs, SiO_2_ NPs, and TiO_2_ NPs in water by DLS and ζ-potential (mV) measurements in water after one month.

Samples	Size (nm)	ζ-Potential (mV)
Au NPs	22 ± 4	−32 ± 4
Ag NPs	21 ± 3	−23 ± 3
SiO_2_ NPs	25 ± 5	−24 ± 2
TiO_2_ NPs	24 ± 16	−21 ± 5

**Table 3:** Conversion of metal concentrations expressed in μM in mass/volume concentrations of NPs used in this study.

NPs	Concentration (μM)	Mass Concentration (mg/mL)	Concentration (μM)	Mass Concentration (mg/mL)
Ag NPs	100	0.011	500	0.054
Au NPs	100	0.020	500	0.098
SiO_2_NPs	100	0.003	500	0.015
TiO_2_NPs	100	0.008	500	0.04

**Table 4:** Characterization of Ag NPs, Au NPs, SiO_2_ NPs, and TiO_2_ NPs in water by DLS and ζ-potential (mV) measurements after the absorption experiments conducted with the three mosses species for 48 h starting from an initial concentration of 500 μM.

Samples	Residual NPs after Absorption by *L. riparium*	Residual NPs after Absorption by *V. ferriei*	Residual NPs after Absorption by *T. barbieri*
	size (nm) ± SD; ζ—potential (mV) ± SD	size (nm) ± SD; ζ—potential (mV) ± SD	size (nm) ± SD; ζ—potential (mV) ± SD
AgNPs	20 nm ± 4; −33 mV ± 2	20 nm ± 2; 33 mV ± 2	21 nm± 3; −33 mV ± 2
AuNPs	19 nm ± 3; −27 mV ± 3	20 nm ± 2; −28 mV ± 2	19 nm ± 4; −33 mV ± 2
SiO_2_NPs	24 nm ± 2; −24 mV ± 4	25 nm ± 2; −25 mV ± 3	24 nm ± 4; −23 mV ± 2
TiO_2_NPs	23 nm ± 7; −22 mV ± 3	24 nm ± 6; −24 mV ± 2	23 nm ± 9; −25 mV ± 2

## Data Availability

Not applicable.

## Sample Availability

Samples of the compounds are not available from the authors.
